# The Impact of the Covid-19 Pandemic on an Integrated Care Programme for Older People with Different Frailty Levels (OPDFL): A Qualitative Study with Service Providers in the East of England

**DOI:** 10.5334/ijic.7703

**Published:** 2024-07-01

**Authors:** Nimra Khan, David Hewson, Gurch Randhawa

**Affiliations:** 1 Department of Psychiatry, University of Oxford, Warneford Hospital, Warneford Ln, Headington, Oxford OX3 7JX, UK; 2 Institute for Health Research, University of Bedfordshire, Hitchin Road, Luton, LU2 8LE, UK

**Keywords:** integrated care, COVID-19, frailty, older people, Luton, England

## Abstract

**Introduction:**

While populations of all ages were affected by the pandemic, older people with frailty had much worse outcomes. The NHS England has mandated identifying and proactively managing older people with moderate and severe frailty in the General medical services (GMS) contract 2017/18. As a result of this policy, an integrated care programme for older people with different frailty levels (OPDFL) was introduced in Luton in 2018 (known as, Luton Framework for Frailty – LFF). This study was conducted to explore the views of service providers in Luton regarding the impact of the COVID-19 pandemic on the implementation of LFF.

**Methods:**

Semi-structured interviews were conducted with service providers in Luton between April 2021 to July 2021. The data were analysed using thematic analysis.

**Results:**

Eighteen service providers took part in the study. Three main themes were identified, the first of which was that proactive and frailty-related health promotion services were halted. Secondly, existing relationships due to the LFF facilitated the implementation of services for care home residents during the pandemic. Finally, participants identified that some of the challenges impacting the delivery of health promotion services were those that affected the health system in general, such as healthcare staff feeling stressed and the centralised decision-making by the government.

**Conclusion:**

The lessons learnt from this study could be useful in managing services for older people with frailty in times of emergencies or epidemics.

## Introduction

Frailty is an age associated long term condition which depletes intrinsic reserves across multiple physiological systems, resulting in a person becoming vulnerable to minor stressors [1]. The recovery from a minor injury for an older person with frailty is slower compared to a person who is non-frail. Frailty is associated with poor health outcomes such as falls and fractures, frequent hospital admissions, Accident & Emergency visits and deaths [2]. Furthermore, managing an individual with frailty costs the NHS much more than an individual with no frailty [3]. NHS England mandated identifying and managing older people with moderate and severe frailty in the General medical services (GMS) contract 2017/18. The GMS contract is a standardised national agreement for General Practitioners (GP) to provide essential medical services. In the UK, a GP is a medical doctor who provides primary medical care and refers patients to hospitals for specialised or urgent services.

As a result of the GMS contact, an integrated care programme, known as the Luton Framework for Frailty (LFF), was introduced to improve services for older people with different frailty levels (OPDFL). It uses the concept of the Kaiser Permanente model (2023), and stratifies older people based on their frailty levels and then offers them integrated care based on their individual frailty level [4]. For example, people who are identified as fit receive a healthy ageing booklet on every birthday after turning 65, which provides guidance on maintaining good health and utilising community resources such as exercise classes. Those who are identified as mildly frail are offered a 12-week free physical activity program after which, they are given the option to continue with the exercises at a discounted cost. There are two pathways for people identified as having moderate and severe frailty: proactive and reactive pathways. The proactive pathway involves identification, using a health-risk management tool, of older adults with moderate or severe frailty by a GP and care coordinator on a monthly basis. Those who are identified as at risk will receive a phone call from a care coordinator who will then conduct a 5M assessment (mobility, matters most, mind, medication, multi-complexity). If the assessment indicates a higher risk of deterioration, the case will be discussed in a multidisciplinary team (MDT) meeting. The MDT include the GP, care coordinator, physiotherapist, practice nurse, falls teams, with other professionals called in as needed. Care plans are then developed based on individual needs, with the care coordinators following up on the care plans. The LFF was mostly integrated at the primary care level, although geriatricians also participated in the MDT meetings. For the reactive pathway, a team comprising a care coordinator, pharmacy technician and community matron identifies older people whose health is declining, and who have either visited the A&E or have been recently discharged from the hospital. They use a MedeAnalytics database (MedeAnalytics Inc, Richardson, TX, USA) to identify these individuals on a daily basis. The care coordinator then conducts a 5M assessment via telephone with these individuals. Anyone who is considered at higher risk of deterioration is further discussed in the MDT meetings (as described above), which occur monthly or bi-monthly, based on the number of older people in the catchment area. Person-centred care plans are then developed and patients are referred to the relevant services [4].

The COVID-19 pandemic had a negative impact globally causing higher morbidity and mortality [5]. In England and Wales, the number of deaths recorded due to COVID-19 until the end of July 2022 was 180,000, which accounts for about 1 in 8 of all deaths [6]. These numbers were unequally distributed among the population with older people being worst affected. Since old age is associated with a decline in immune system functioning, older people were more likely to develop severe symptoms with the infection. For example, in England and Wales at ages 80 to 84 the mortality rate was 6.5 times higher than at ages 65 to 69, and 57 times higher than at ages under 65 [6].

Older people with frailty were also at higher risk of developing severe illness with COVID-19. The LFF was introduced in late 2018 and was in its early implementation phase when the COVID-19 pandemic started. It was widely reported that the COVID-19 pandemic had disrupted the existing service provision with much of the preventative work halted to manage the response to the pandemic [7,8]. This study was part of the overall evaluation of the LFF.

The aim of this study was to explore the views of service providers regarding the impact of the COVID-19 pandemic on the implementation of an integrated care programme for OPDFL in Luton.

## Methods

### Study Setting

This study was conducted in Luton, a town situated in the southeast of England with a population of 225,300 [9]. Luton has high rates of deprivation and is ranked as the 70^th^ most deprived area out of the 327 local authorities in England [9]. There are health inequalities across the socioeconomic gradient, while health indicators of people in Luton are worse than the national average. Among those residents identified as most deprived, life expectancy is 10.4 and 6.3 years less for men and women, respectively [10]. Luton is also an ethnically diverse town with over 55% of the population belonging to the Black, Asian, and Minority Ethnic groups [9].

### Design

A qualitative study with semi-structured interviews was conducted from April 2021 to July 2021. Qualitative research provides rich data that improves the understanding of people’s experiences [11]. The use of qualitative methods in evaluation studies is growing, as it enables answers to be sought to questions such as: how decisions are made, how an intervention is implemented, and how interventions get affected by contextual factors [12]. This study is part of NK’s PhD project, which was underpinned by the Chronic Care Model (CCM). The CCM is a framework used to describe integrated care interventions that focuses on six components, which are healthcare organisation, delivery system design, health information systems, self-management support, community resources, and decision support.

### Participants

The participant recruitment process utilised the purposive sampling technique, which is employed to recruit individuals who possess experience and knowledge about the phenomenon being studied. The LFF program’s design and delivery included stakeholders from various sectors, such as primary and secondary care, as well as the voluntary sector. The Luton Clinical Commissioning Group (CCG) identified potential participants who were key professionals for LFF. The CCG then sent introductory emails to all the professionals, including the authors’ (NK) email address, which participants could contact if they wished to take part in the study. Individuals who responded to the email were included in the study, participants from both statutory and non-statutory organisations. The participants included different stakeholder groups, such as geriatricians, GPs, nurses, managers and commissioners. The inclusion criteria required the participants to have a strategic engagement in planning and managing care processes and involvement in implementing the LFF.

The sample size for qualitative studies is usually smaller than for quantitative studies [13]. It should be large enough to understand the phenomenon under investigation, but small enough that an in-depth case-oriented analysis could be carried out [14]. In total 22 participants were invited to take part, however only 18 of the stakeholders invited participated. One of the reasons for not having all the stakeholders take part was that the study took place during COVID-19, and those involved in health services delivery were generally under huge work pressures. To ensure that professionals could take part, the study was delayed by 4 months.

### Data Collection

Semi-structured interviews were conducted as they are a suitable method to explore the beliefs, motivations, experiences, and views of respondents on a topic [15]. The topic guide for this study was based on existing literature [16,17,18,19]. An example of the prompts given were: In terms of the services provided to OPDFL, what went well during COVID-19? What did not go so well? Due to COVID-19 restrictions, 16 interviews were conducted on Teams and 2 were on the telephone.

### Analysis

Data were analysed using Braun and Clark’s six-step thematic analysis approach [20]. Thematic analysis is an interpretive approach to identifying patterns in the data to explain a phenomenon [21]. The findings of such analysis provide themes instead of an explicit theory. A theme finds some important information in the data related to the research question or captures some form of the pattern or meaning within the dataset [22]. The audio-recorded data files were transcribed verbatim. To familiarise with the data, the transcripts were read and reread, and notes were taken to capture initial impressions. Open coding was then used to organise data into small chunks of meaning. The codes that fitted together and addressed the research question were described as a sub-theme or a theme. All themes were reviewed, and all relevant data was compiled for each sub-theme or theme. The entire data analysis was an iterative process, with researchers meeting regularly throughout the analysis. The data was primarily analysed by NK, while DH also coded a sample of the data. Any disagreements were discussed in group meetings with all the researchers, and a consensus was reached. After the first 13 interviews, no new themes were generated in the remaining interviews, indicating data saturation.

### Ethics

As this study was a service evaluation, it did not require NHS ethics approval. Moreover, the Health Research Authority decision-making checklist was applied, and it confirmed that the study does not require NHS ethics approval. Nevertheless, ethics approval was obtained from the Institute of Health Research at the University of Bedfordshire (IHREC953).

## Results

The18 service providers who took part in the semi-structured interviews were from diverse professional backgrounds (Table 1). Three themes were identified regarding participants’ beliefs about the impact of COVID-19 pandemic on the implementation of an integrated care programme for OPDFL:

- Proactive and frailty related health promotion services were halted during COVID-19,
- The LFF programme facilitated delivery of services for care home residents, and
- Issues experienced by stakeholders across the health system in the COVID-19 response.

**Table 1:** Job description of the participants.


PARTICIPANTS	JOB DESCRIPTION (PROFESSIONAL DISCIPLINE)

**P1**	Senior Leadership Role (Public Health Professional)

**P2**	Senior General Practitioner

**P3**	Senior Manager of a Service (Nurse)

**P4**	Senior Manager of a Service (Nurse)

**P5**	Pharmacist

**P6**	Senior Manager of a Service (Nurse)

**P7**	Senior Geriatrician

**P8**	Team Lead for a Service (Management background)

**P9**	Team Lead for a Service (Occupational therapist)

**P10**	Senior Leadership Role (Physiotherapist)

**P11**	Senior Pharmacist

**P12**	Pharmacist

**P13**	Commissioner

**P14**	Senior Commissioner

**P15**	GP

**P16**	Senior GP

**P17**	GP

**P18**	Senior GP


### Theme 1: Proactive and frailty related health promotion services were halted during COVID-19

The LFF offered a free 12-week exercise program for older people with mild frailty. However, when the COVID-19 pandemic started, this program was stopped. Participants mentioned that other exercise programs were also stopped, and that older adults were generally told to shield. While they supported that older people should be shielding, they also thought this might have had a significant impact on the health of older people with frailty. Some participants pointed out that the Government or local health authorities did not provide any guidelines to OPDFL on how to stay active during the lockdown. This lack of guidance might have negatively affected OPDFL’s mental and physical health.

*“So, I think, elderly people have generally been asked to shield, and they will have done that, and taken the consequences of that, in a way that we may not know the full impact of that social isolation and the difficulties of being stuck at home. The government’s probably mean right to ask people to be shielded…however, there hasn’t been so much information from the governments about how to try and stay healthy in lockdown”* (P16, Senior GP).

*“I think it would have been good to have mental health support for those who actually sort of shield and stay indoors for this length of time. I think a lot of people got very, very lonely and depressed. And I think that was a detrimental effect on a lot of old folks”* (P2, Senior GP).

One participant mentioned the negative impact of the isolation caused by COVID-19 restrictions on older people’s mental health, which had led to a rise in suicides among those who are 70 years and older. Upon seeing this, training was introduced to identify people with deteriorating mental health early on and enable collaboration with other health professionals.

*“We have observed an escalation in the number of suicides amongst the elderly people over the last twelve months…perhaps due to the COVID-19 related isolation…these people were never known to mental health services before…we are offering training to create awareness of impact of current circumstances or all circumstances on mental health so our GP colleagues or our matrons or local authority could recognise that very, very early and try to get those people the support before it goes wrong”* (P10, Senior Leadership Role).

Participants reported that the proactive pathway for older people with moderate and severe frailty was also stopped.

*“Covid-19 has made everything reactive… things like routine reviews and all the proactive work has been put on hold”* (P8, Team Lead for a Service).

*“Frailty clinics with the practices that have now all gone”* (P4, Senior Manager for a Service).

### Theme 2: The LFF programme facilitated delivery of services for care home residents

The participants stated that the care pathways and relationships established in Luton, as part of the LFF, and the overall commitment to offering services for OPDFL, facilitated the delivery of the COVID-19 response. For instance, proactive anticipatory care plans were developed for moderate and severely frail older people.

*“…in the early days of Covid-19 because we can see that people might be at risk of becoming severely ill with Covid-19 there was quite a significant push to advanced care planning and treatment escalation plan for our care home patients and also for some of our very severe patients just to think through what would you want if you got Covid-19, would you want to be actively treated, would you want to be admitted to hospital”* (P18, Senior GP).

The Enhanced Health in Care Homes (EHCH) initiative had mandated the alignment of care homes with general practices between 2020 to 2023. In Luton, all care homes were aligned with general practices, and GPs conducted weekly multidisciplinary team (MDT) meetings to identify any issues faced by residents. Although the EHCH initiative was not directly part of LFF, the existing integrated way of working and good working relationships across different organisations due to LFF facilitated the rapid implementation of EHCH. This change supported care home staff in better managing their residents, while also helping to manage COVID-19 outbreaks in care homes.

*“Some of the things that NHS England mandated e.g. aligning care homes with the GP practices and conducting MDT meetings…it wasn’t actually difficult for us as we were already conducting MDT meetings for our frail patients…those relationships were there”* (P14, Senior Commissioner).

*“In Luton we had good working relationships…professionals across organisations and healthcare levels knew each other and were part of the Multidisciplinary teams”* (P5, Pharmacist).

*“…The other thing we did was to align the care homes to the GP practices…that meant you could be more systematic in terms of thinking through, what care planning needs were of particular people… whereas before…it was very hard for the home to coordinate things…”* (P16, Senior GP).

### Theme 3: Issues experienced by stakeholders across the health system in the COVID-19 response

Participants highlighted common issues experienced by health providers and stakeholders across the health system. For instance, healthcare staff were feeling stressed due to concerns about their own safety and that of their families, as well as the emotional strain of witnessing so many patients seriously ill and dying without their loved ones beside them.

*“…A lot of people in my team asking, am I going to get sick, am I going to die…putting themselves at risk, seeing the impact on our patients, again quite distressing…”* (P1, Senior Leadership Role).

One participant described the staff were ‘*fearful*’ and had *‘anxiety of catching the virus and taking it to their families’* (P18, Senior GP).

Some participants expressed their dissatisfaction with the Government’s handling of the pandemic. They have pointed out that the Government announced the implementation of covid-19 specific measures on a national level without considering the local infrastructure needed to implement it properly. The translation of some policies was particularly challenging due to the lack of laboratory infrastructure and professional capacity. For example, COVID-19 testing of all care home workers every week or provide iPads in all care homes.

*“…all of these decisions were being made by the Prime Minister and their cabinet and the only time the care homes, or we knew was when they were announced to the nation…Every care home worker had to be tested once a week, how do you do that?…I think they didn’t understand the numbers…they must be having mountains of these swabs…”* (P13, Commissioner).

The majority of participants felt that stopping care home visits was a very painful experience for both residents and their families. They also felt that there should have been an alternative to visiting, such as digital access.

*“…the biggest challenge has been care home visiting…a very painful experience for a lot of relatives and care home patients not being able to see their relatives…relatives were very concerned about them deteriorating…maybe they (the Government) needed to push the digital access”* (P16, Senior GP).

## Discussion

This study presents the perspectives of service providers on the effect of the COVID-19 pandemic on the implementation of an integrated care program for OPDFL. According to the service providers, the pandemic led to the cessation of proactive and health promotion services for OPDFL. The exercise programme for older people with mild frailty and the proactive care pathway for older people with moderate and severe frailty were stopped in Luton. Participants highlighted that although shielding and staying safe was important, the detrimental impact of the pandemic on the physical and mental health of the elderly was not fully assessed. Furthermore, no guidance was provided at the national or local level on how to maintain good health during the shielding periods. It was reported that mental health of elderly in Luton was deteriorating leading to increased suicides. Thus, healthcare staff were trained to spot early signs of mental health deterioration.

Studies have shown that the shielding policy for older people during the COVID-19 pandemic has had a negative impact on their mental and physical health [23,24]. Baily et al. (2021) found that a majority of older individuals experienced a decline in mental and physical well-being, as well as reduced physical activity, compared to the period before the restrictions were imposed [25]. Additionally, many people reported feeling lonely on a regular basis. The authors suggested that clear policies and advice prioritising physical activity, social engagement, and loneliness management should be created for older people.

Participants reported that the implementation of the enhanced health in care homes (EHCH) program in Luton was not challenging due to the pre-existing relationships and integrated way of working among stakeholders. The stakeholders utilised their existing relationships to expedite the implementation of the EHCH initiative. Additionally, they were able to conduct anticipatory care planning for the elderly with severe frailty in the community with ease.

While less is known about the experience of implementation of EHCH during COVID-19, it is well-known that established relationships facilitate implementation of integrated care interventions. For instance, a study looking at barriers and facilitators to the integration of complementary and alternative medicine for musculoskeletal and mental health within the NHS [26] that good working relationships were a key facilitator for integration. Other studies have also found that strong working relationships and good planning facilitate integrated working [27,28]. In a study by MacInnes et al. (2020), the working climate of teams involved in implementing integrated care programmes for older people in thirteen sites across European countries was explored. They found that, among other factors, strong relationships, higher levels of commitment, and motivation were important drivers for collaboration [28].

Service providers in this study reported that COVID-19 had a negative impact on the NHS staff, who were stressed. They described that the number of people dying was unprecedented, and that staff were anxious about getting infected and potentially infecting their families. Other studies assessing the impact of the pandemic on healthcare staff have shown that healthcare providers have experienced poor mental health and increased stress during the pandemic response [7,8]. Service providers generally viewed the role of the Government during the pandemic with scepticism. They described that many decisions were made at the top, without considering local capacity, while they also found the Government’s messaging to often be confusing. The UK government’s response to COVID-19 has been criticised for being overly centralised; for instance, the Government started purchasing services directly from the private sector such as the test and trace but did not use the knowledge of existing professionals at the local level who were in a better position to perform this role [29,30]. Participants in this study reported the painful experiences of residents and their families over the no visitation policy. Other studies have also described similar experiences of care home residents and suggested that there should have been alternative digital options for families and residents to stay in contact [31,32,33].

One of the limitations of this study is that it reflects upon the views of service providers and no insights of service users were obtained; an issue which a future study should address. Nevertheless, this was the first study to explore the views of services providers involved in delivering integrated care for OPDFL in one site in England. Findings from this research offer insights to the policy makers and service providers nationally and locally about what worked well and what could have been improved in the COVID-19 response.
